# Body cell mass evaluation in critically ill patients: killing two birds with one stone

**DOI:** 10.1186/cc13852

**Published:** 2014-05-01

**Authors:** Enrico Fiaccadori, Santo Morabito, Aderville Cabassi, Giuseppe Regolisti

**Affiliations:** 1 Department of Clinical and Experimental Medicine, Acute and Chronic Renal Failure Unit, Parma University, 43126 Parma, Italy; 2 Department of Nephrology and Urology, Hemodialysis Unit, 'Sapienza' University, 00161 Rome, Italy

## Abstract

Body cell mass (BCM) is the metabolically active cell mass involved in O_2_ consumption, CO_2_ production and energy expenditure. BCM measurement has been suggested as a tool for the evaluation of nutritional status. Since BCM is closely related to energy expenditure, it could also represent a good reference value for the calculation of nutrient needs. In a recent issue of *Critical Care*, Ismael and colleagues used bioelectrical impedance analysis parameters and anthropometric variables to evaluate BCM in patients with acute kidney injury, before and after a hemodialysis session. The results of this study suggest that BCM is relatively insensitive to major body fluid shifts, a well known factor interfering with nutritional evaluation/monitoring and energy need calculations in the ICU. Thus, BCM seems to be a more 'stable' nutritional variable, as it is apparently less influenced by non-nutritional factors. The results of this paper emphasize the need to identify biologically sound parameters for nutritional status evaluation and energy need calculation in critically ill patients; in this regard, BCM could fulfill these expectations.

## 

In a recent issue of *Critical Care*, Ismael and colleagues [1] reported the results of a study on 31 hemodynamically stable patients with acute kidney injury requiring hemodialysis and able to tolerate ultrafiltration rates of ≥5% body weight per session (mean weight loss of 3.8 kg). They derived intra- and extracellular water volumes from low- and high-frequency resistances measured by multi-frequency bioelectrical impedance analysis (BIA) of body compartments, before and after hemodialysis. Moreover, they estimated body cell mass (BCM) from BIA parameters and anthropometric variables, based on a recently developed regression equation specific for ICU patients [2]. The investigation aimed at evaluating the consistency and clinical relevance of the current model for BCM calculation in case of massive changes in the external fluid balance. The results of this promising study suggest that estimated BCM is relatively insensitive to major body fluid shifts, a frequent problem among critically ill patients, and also a well known factor interfering with nutritional evaluation and monitoring in this clinical setting. Thus, BCM seems to be a more 'stable' nutritional variable, as it appears less influenced by non-nutritional factors.

Many factors may significantly interfere with the measurement and interpretation of the classical nutritional variables in the ICU, especially when rapid changes of fluid balance and/or renal function derangements are present [3].

At the same time, the precise definition of energy needs, when the gold standard of indirect calorimetry is not available, still relies on estimations obtained from predictive formulas or on calculations using a fixed amount of calories per kilogram of body weight [4], with the latter being not always measured, and often heavily affected by rapid modifications of fluid balance.

Critically ill patients may have wide variations in energy requirements [4,5], thus being at high risk for both underfeeding and overfeeding. Therefore, the issue of administering the right amount of calories at the right time to these patients has gained major attention [6,7]. Indeed, recent data suggest that individual tailoring of calorie supplementation in terms of both timing [8] and intake [9,10] could impact significantly on the effects of nutritional support on patients’ prognosis.

BCM is the metabolically active component of fat-free mass, and represents the cell mass actually involved in O_2_ consumption, CO_2_ production and energy expenditure [11]. It is negatively affected in the course of critical illness, and its measurement has been suggested as a tool for the evaluation of nutritional status in these patients [11,12]. Moreover, since it is closely related to energy expenditure, BCM could also represent a good reference value for the calculation of nutrient needs.

BCM can be directly measured by isotope dilution methods (that is, ^40^ K), or estimated by predictive equations [2]. However, the gold standard methods based on radioactive tracer techniques are not amenable to routine use in the ICU. On the other hand, bedside estimation of BCM, essentially using equations based on BIA of body compartments, could represent a simple, non-invasive and repeatable tool for nutritional status evaluation in critically ill patients.

The results of the paper by Ismael and associates [1] emphasize the need to identify biologically sound parameters for nutritional status evaluation and energy need calculation in critically ill patients; in this regard, BCM could fulfill these expectations.

Encouraging results have been obtained recently in other clinical settings when interventions were tailored based on BIA of body composition [13]. A number of open issues remain, however, and the overall impression is that more data should be collected before this promising approach can be applied routinely in ICU patients. Undoubtedly, by choosing short hemodialysis sessions, the authors exploited a simple clinical model of rapid fluid balance changes. However, the need to use high net ultrafiltration rates inevitably selects a relatively more healthy population of hemodynamically stable ICU patients. Moreover, a recent study that investigated the intrinsic error of the BIA methods for estimating body fluid volume/composition in chronic dialysis patients demonstrated that such estimates can be consistent at a population level, but not always at the individual level due to wide limits of agreement [14]. Within this conceptual framework, it has been also correctly observed that the prediction error of methods based on BIA of body compartments is the sum of many errors, namely the impedance measurement error, the regression error, the intrinsic error of the reference method, the electric volume model error, and the biological variability among subjects [15].

## Conclusion

In the selected population of ICU patients of their study, the authors’ approach seems to be physiologically sound and effective. Thus, although their results cannot be immediately translated to the complex and dynamically changing clinical setting of the ICU, they are surely encouraging and should prompt further investigations on this innovative approach that could possibly kill two birds with one stone.

## Abbreviations

BCM: Body cell mass; BIA: Bioelectrical impedance analysis.

## Competing interests

The authors declare that they have no competing interests.
